# Supplementary figures and images for: Ectopic expression of WRINKLED1 in rice improves lipid biosynthesis but retards plant growth and development
Source: PLoS One. 2022 Aug 19;17(8):e0267684. doi: 10.1371/journal.pone.0267684 (PMC9390937; doi:10.1371/journal.pone.0267684)

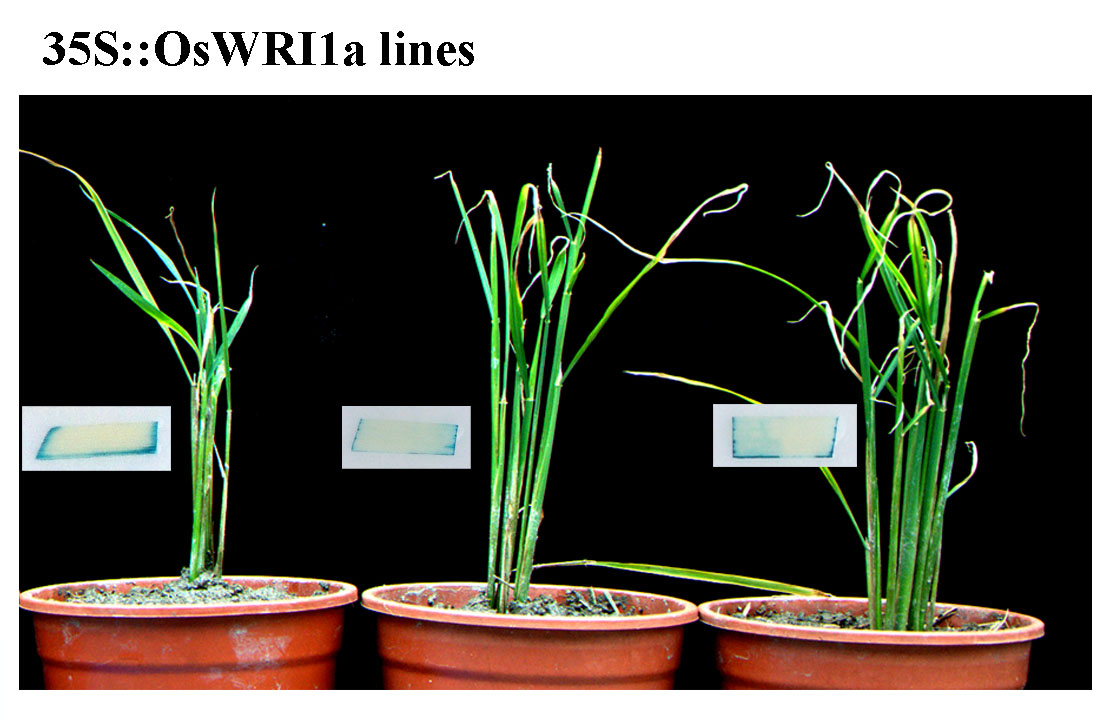

Supplement: S1 Fig — Putative transformants were selected by detecting GUS reporter gene expression by means of a GUS staining assay. The (three-month-old) seedlings exhibited dwarfism, the upper parts of the laminae of most leaves were chlorotic and curly, and these leaves died more rapidly than those of wild-type plants. (JPG) [file pone.0267684.s002.jpg]

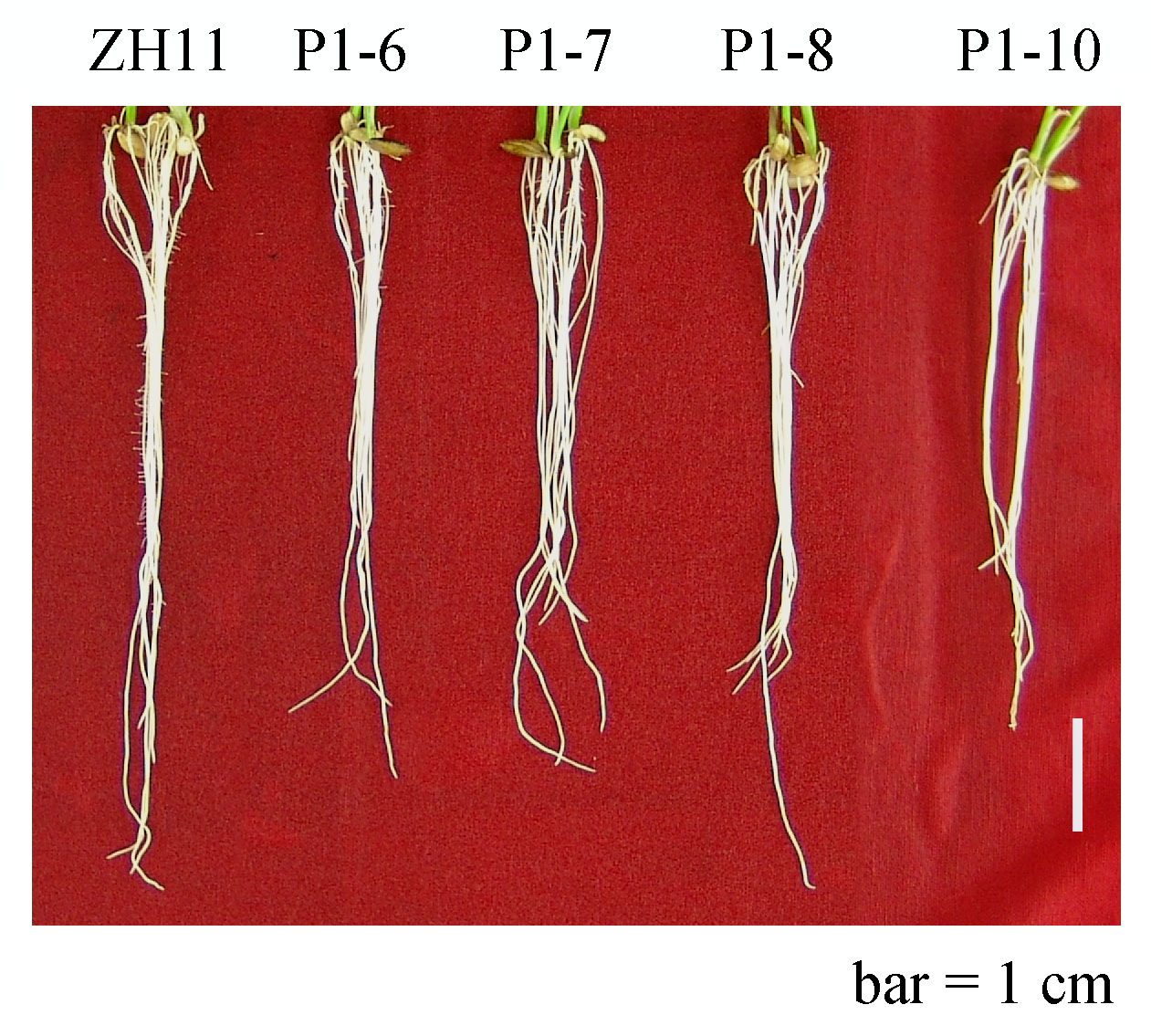

Supplement: S2 Fig — (JPG) [file pone.0267684.s003.jpg]

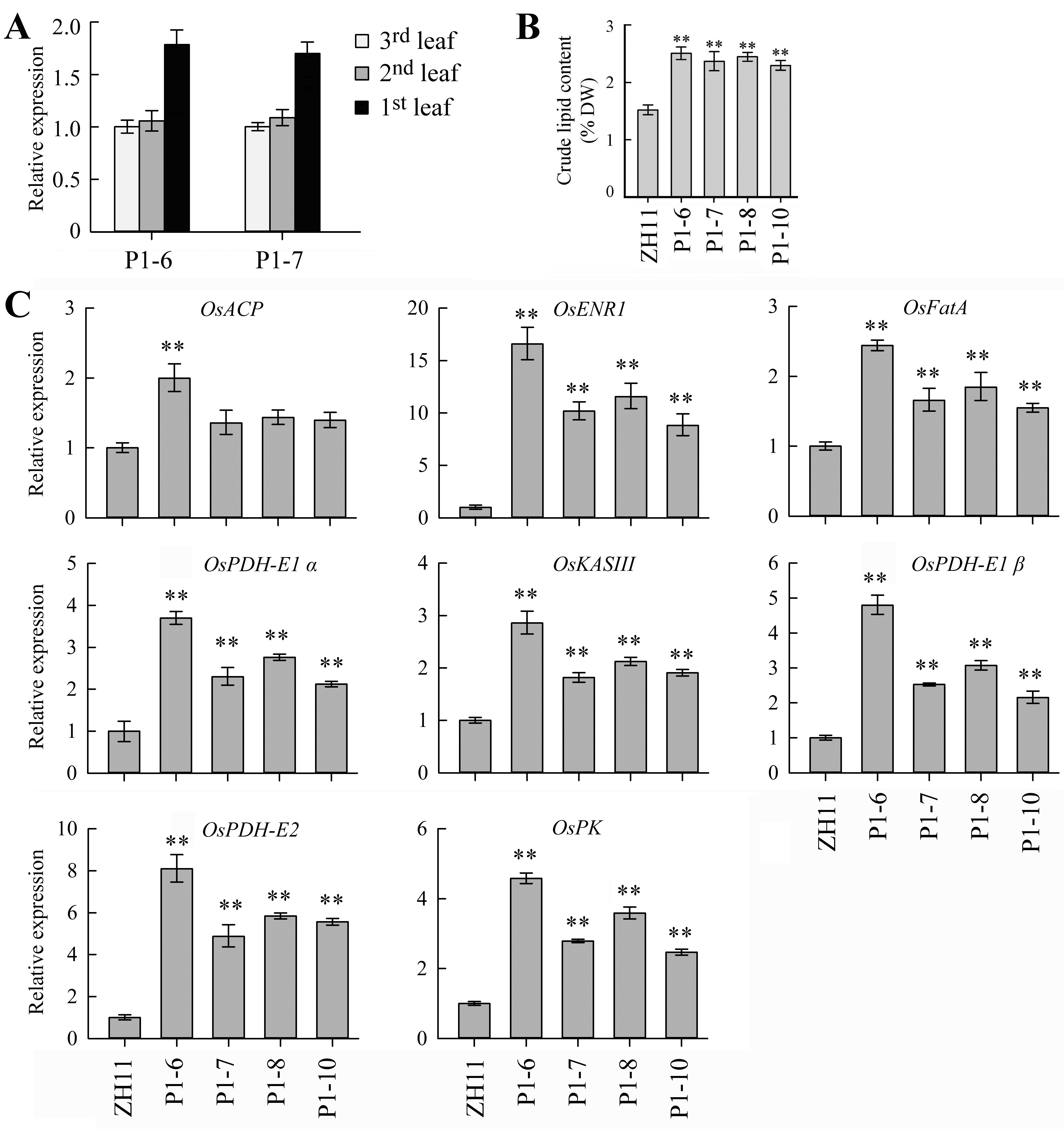

Supplement: S3 Fig — (A) The levels of OsWRI1 transcripts in flag leaf (1st leaf), the second (2nd leaf) and the third (3rd leaf) leaves at the stage of the flag leaf was about 10 cm in length in two PBt2P1::OsWRI1a transgenic lines of P1-6 and P1-7. (B) The crude lipid content in leaves of heading stage plants. Values represent means of n = 6 ± SD (Duncan test: **, P < 0.01). DW = dry weight. (C) Expression of genes predicted to encode proteins involved in the later stages of glycolysis and fatty acid biosynthetic pathways in leaves. Total RNA was isolated from the second leaves of plants at heading time. The experiment included three biological replicates, each with two technical replicates. Values represent means of n = 6 ± SD (Duncan test: **, P < 0.01). (JPG) [file pone.0267684.s004.jpg]

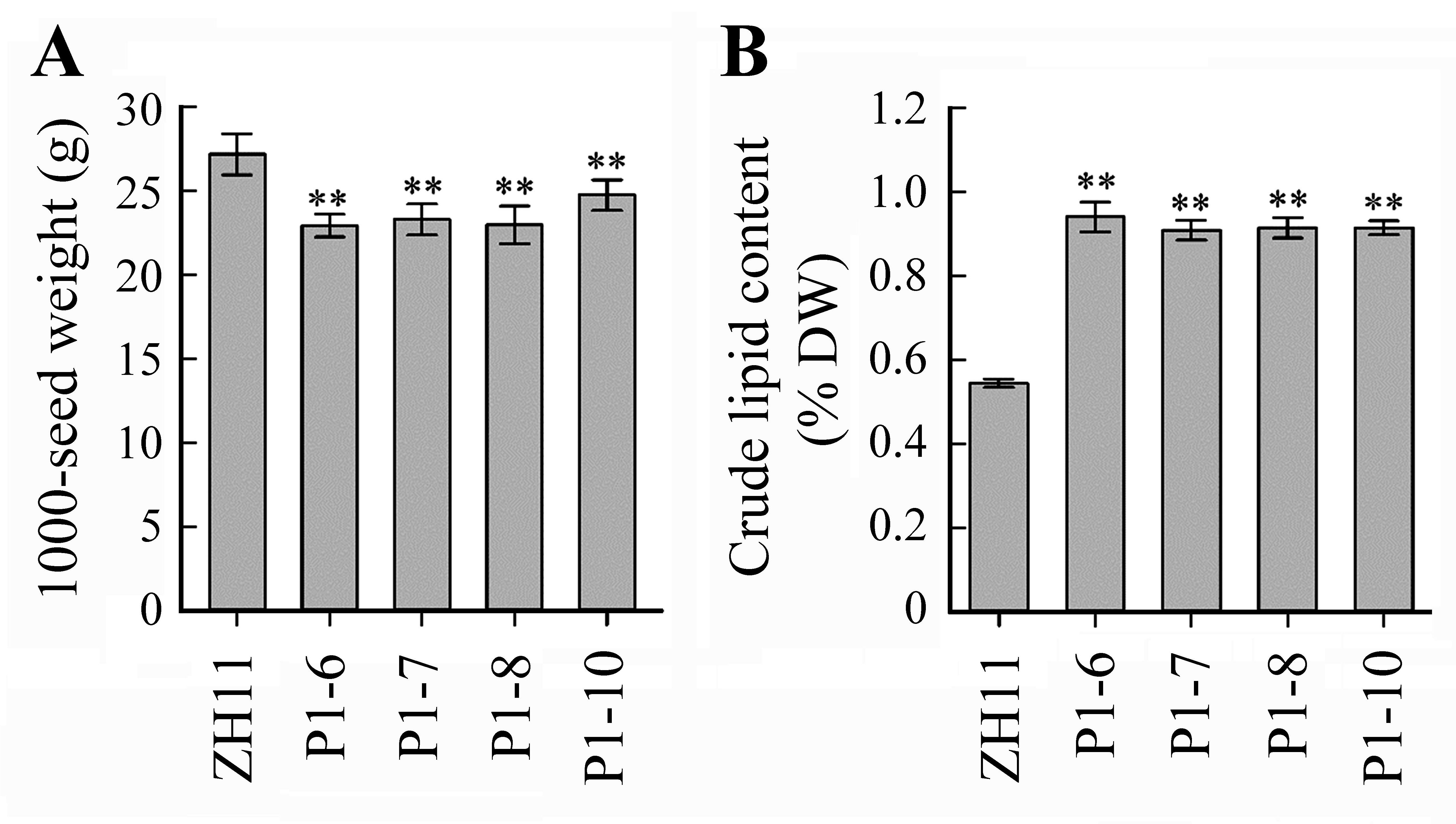

Supplement: S4 Fig — (A) The 1000-seed weight. Seed weights were calculated by randomly selected seeds. The mature seeds were dried under 37°C in an oven for three days. Values represent means of n = 3 ± SD (Duncan test: **, P < 0.01). (B) The crude lipid content in endosperm of mature seeds. Values represent means of n = 6 ± SD (Duncan test: **, P < 0.01; *, P < 0.05). DW = dry weight. (JPG) [file pone.0267684.s005.jpg]
